# Infrequent delayed controlateral remote cerebellar hemorrhage after supratentorial craniotomy in adult patient: a case report

**DOI:** 10.1016/j.radcr.2022.09.051

**Published:** 2022-10-06

**Authors:** Mehdi Borni, Souhir Abdelmouleh, Mohamed Ghorbel, Amal Ben Belgacem, Mohamed Zaher Boudawara

**Affiliations:** Department of Neurosurgery, UHC Habib Bourguiba, Sfax, Tunisia

## Abstract

Remote cerebellar hemorrhage as a rare complication of supratentorial surgery was already first described in the 1970s by Yasargil. Its incidence ranges from 0.2% to 0.4% after supratentorial craniotomies. Although its incidence is low, the volume of reports with remote cerebellar hemorrhage in the literature has been growing in recent times. The authors report here a new case of a controlateral remote cerebellar hemorrhage after 24 hours of supratentorial craniotomy for a solitary brain metastasis of a pulmonary adenocarcinoma in a 59 year-old male patient with unbalanced high blood pressure.

Supratentorial craniotomy, Remote cerebellar hemorrhage, CT scan

## Introduction

Remote cerebellar hemorrhage (RCH) as a rare complication of supratentorial surgery was already first described in the 1970s by Yasargil [1]. Its incidence, according to Konig et al. [2], ranges from 0.2% to 0.4% after supratentorial craniotomies. This complication affects cranial and spinal surgery and both adult and pediatric patients [3,4]. Although classically it was believed to be an entity observed exclusively in patients undergoing frontotemporal craniotomies, it is currently known that it does not have a specific relationship with the type of surgery performed, detecting this complication in a wide variety of neurosurgical procedures [5], [6], [7]. Patients with RCH are usually between 30 and 60 years old, however cases have been reported in patients younger than 10 years and older than 80 [6]. Although the incidence of this complication is low, the volume of reports with remote cerebellar hemorrhage in the literature has been growing in recent times [8,9].

The authors report here a new case of a controlateral remote cerebellar hemorrhage after 24 hours of supratentorial craniotomy for a solitary brain metastasis of a pulmonary adenocarcinoma in a 59 year-old male patient with unbalanced high blood pressure.

## Case report

**Fig. 1:**
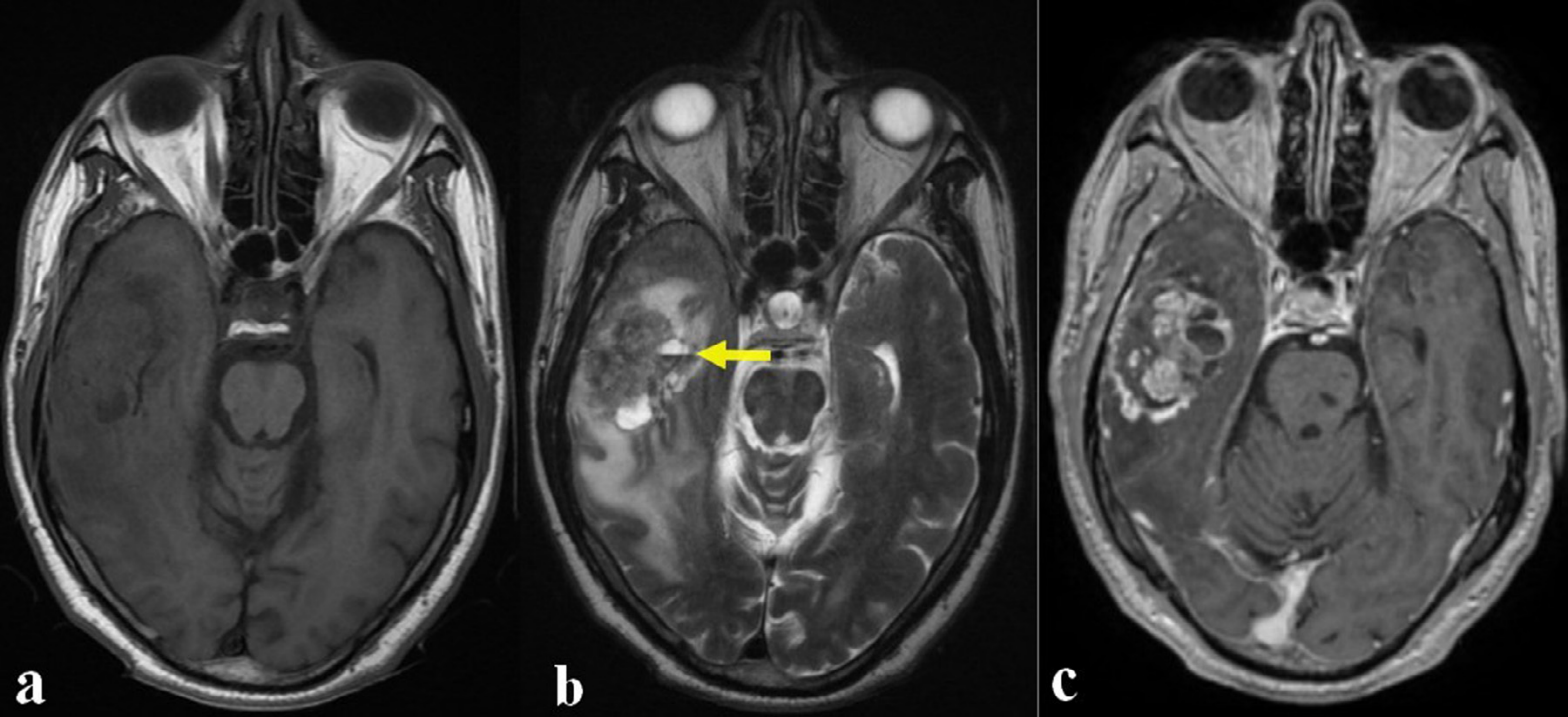
Brain MRI in axial plane showing a right temporal tumor, in solid and cystic geographical map of polycyclic contours. Its fleshy portion had an isosignal on T1-weighted sequences (**a**) and a heterogeneous signal on the T2-weighted sequences (**b**). This lesion developed areas of necrosis and is the seat of a liquid liquid level (blood liquid) (**b**; yellow arrow). Gadolinuim chelate enhancement was heterogeneous (**c**).

A 59-year-old male patient known to have unbalanced high blood pressure with no other particular medical or surgical history was admitted to our department of neurosurgery for progressive onset of raised intracranial pressure made of constrictive headache and bilateral blurred vision associated to recent lower left limb heaviness. The patient as his family denied any prior traumatic or epileptic event. Upon examination, our patient was fully awake and apyretic. His neurocognitive function assessment revealed a slurred speech made of poor pronunciation of words, mumbling, and change in speed during a conversation. The rest of the neurological exam showed a predominantly crural left hemiparesis not exceeding 4/5. An immediate brain magnetic resonance imaging (MRI) (Fig. 1) showed a right temporal tumor, in solid and cystic geographical map of polycyclic contours. The fleshy portion had an isosignal on T1-weighted sequences and a heterogeneous signal on the T2-weighted sequences. This lesion developed areas of necrosis and is the seat of a liquid liquid level (blood liquid). Gadolinuim chelate enhancement was heterogeneous with significant hyperperfusion. The whole exerted a mass effect on the right homolateral ventricle with a midline shift of 7 mm. This was associated with a minimal uncal herniation.

The patient underwent an urgent gross total tumor resection through a frontotemporal craniotomy. Intraoperatively he presented a significant intraoperative systolic arterial hypertension managed by vasodilator drugs, which made it possible to continue excision in complete safety.

**Fig. 2:**
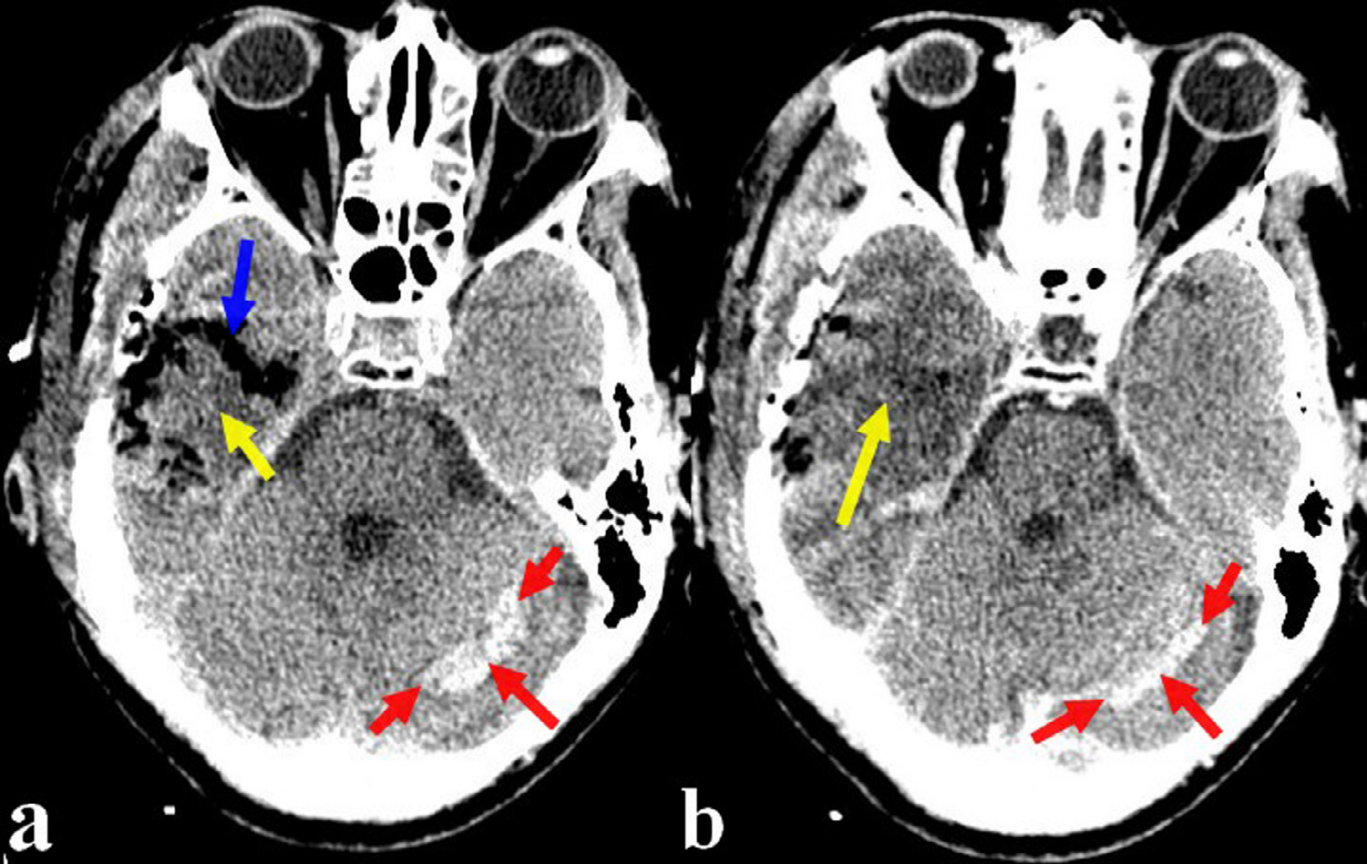
Brain CT scan in axial plane and parenchymal window before (a) and after enhancement (b) performed 24 hours postoperatively showing a postoperative oedemato-hemorrhagic change (**a, b**; yellow arrows) without obvious radiological signs of residual tumor associated with right temporal pneumocephalus (**a**; blue arrow). The whole was associated with a left cerebellar hematoma of 11 × 13 mm in diameter (**a, b**; red arrows) without a mass effect on the fourth ventricle.

The postoperative course was fortunately uneventful. The enhanced postoperative control computed tomography (CT) scan (Fig. 2) performed 24 hours later showed a postoperative edemato-hemorrhagic change without obvious radiological signs of residual tumor associated with right temporal pneumocephalus. The whole was associated with a left cerebellar hematoma of 11 × 13 mm in diameter without a mass effect on the fourth ventricle.

The histopathological examination of the specimen concluded to a cerebral infiltration by an adenocarcinoma whose immunohistochemical profile was in favor of pulmonary origin.

The patient continued to improve his neurological deficit and was subsequently discharged from our department 5 days later and transferred to the radiotherapy department for further investigation and further management.

## Discussion

As noted above, RCH was initially described in surgeries at the supratentorial level [2,[5], [6], [7]] with reports of unruptured [9], [10], [11] and ruptured aneurysm clipping surgery [5,^9^,^10^,^12^,13], temporal lobectomies [14,15], resection of meningiomas [9,16], suprasellar tumors [17] and other brain tumors [4,^5^,9], evacuation of intracerebral hematomas [9], subdural [9] and arachnoid cysts [5], excision of cavernous malformations [5], diversion of cerebrospinal fluid (CSF) [18] and open head trauma by firearm [5]. Among the risk factors found to date, only intraoperative systolic arterial hypertension as it was seen in our patient, yet the presence of intraoperative arterial hypertension is only reported in the literature in 22% of cases [19]. Hypotensive infusion (esmolol hydrochloride), as well as the use of acetylsalicylic acid in the 7 preoperative days were also reported as major risk factors [20].Our patient did not receive any of these drugs before surgery. Intraoperative CSF loss or the use of subgaleal drainage has been suggested as risk factors, but this has not been statistically proven [8].

Although the physiopathology of RCH is not elucidated, most authors agree on its venous origin, since per- or postoperative CSF loss would play an important role [8,16]. On the one hand, the increase in venous pressure (loss of CSF and positional factors) would predispose to venous infarctions and rupture of the subtentorial bridging cerebellar veins by traction secondary to CSF drainage would favor venous hemorrhages [13].

Some risk factors are debated according to authors: peroperative position (neck hyperextension, internal jugular compression by lateral head deviation, prone position [21]). Moreover, all procedures which result in an increase in cerebral hypotonia or increased risk of bleeding should be considered as risk factors for RCH and should therefore be known and avoided [20].

RCH mainly occurs within the first 10 postoperative hours [16]. In our case, we diagnosed radiologically the RCH within 24 hours postoperatively on the routine postoperative CT scan as our patient was symptoms free. Decreased level of consciousness and prolonged awakening from anesthesia are the most common forms of presentation, however, in many cases [6] patients do not present symptoms like our patient. It is also frequently associated with seizures [20]; the presence of other focal symptoms is usually attributable to other intercurrent lesions [20].

The typical imaging pattern is a hemorrhage into the subarachnoid space of the cerebellar leaf (zebra sign) [13], with parenchymal or vermian cerebellar hemorrhage also found [20]. Presentation may be unilateral (ipsilateral or contralateral to surgery as it was seen in our patient) or bilateral [16]. In half of the cases, supratentorial hemorrhage may take place together [20]. Magnetic resonance imaging (MRI) may show residues of hemosiderin accumulation foci in the superior cerebellar folia, subarachnoid hemorrhage, diffuse hemorrhage in the cerebellar tonsils, vermis, or within the fourth ventricle [22].

The treatment could be expectant as in the case of our patient; however, this may require surgical measures such as placement of external CSF shunts and/or posterior fossa decompression [23]. The therapeutic decision in each patient is a case-by-case decision considering the same factors as in cerebellar hemorrhages of other etiologies [9,16].

Although cerebellar hemorrhage is associated with 8.4% morbidity [9] and 14.5% mortality [16], the prognosis is generally better than spontaneous cerebellar hemorrhages [9]. Thirty-two point three percent of patients remain without permanent neurological deficits; the patient's age and the amount of bleeding are the only prognostic factors identified [16].

## Conclusion

From a functional point of view, RCH remains a rare complication of supratentorial neurosurgery which must be suspected in case of postoperative complications even when it is a minimally invasive surgery. Diagnosis is confirmed by cerebral imaging and its prevention requires controlled depletion of CSF during surgery and maintenance of normal perioperative blood pressure. Its treatment is often symptomatic, allowing most often favorable outcomes.

## Patient consent

Written informed consent was obtained from the patient for publication of this case report and any accompanying images.
